# Introduction of *Mycobacterium ulcerans* disease in the Bankim Health District of Cameroon follows damming of the Mapé River

**DOI:** 10.1371/journal.pntd.0008501

**Published:** 2020-09-04

**Authors:** Koen Vandelannoote, Gerd Pluschke, Miriam Bolz, Martin W. Bratschi, Sarah Kerber, Timothy P. Stinear, Bouke C. de Jong

**Affiliations:** 1 Department of Biomedical Sciences, Institute of Tropical Medicine, Antwerp, Belgium; 2 Department of Microbiology and Immunology, The University of Melbourne at the Doherty Institute for Infection & Immunity, Melbourne, Australia; 3 Molecular Immunology, Swiss Tropical Institute, Basel, Switzerland; 4 University of Basel, Basel, Switzerland; Colorado State University - Global Campus, UNITED STATES

## Abstract

Buruli ulcer (BU) is an emerging ulcerative skin disease caused by infection with *Mycobacterium ulcerans*. Efforts to control its spread have been hampered by our limited understanding of *M*. *ulcerans* reservoirs and transmission, and the factors leading to the emergence of BU disease in a particular region. In this report we investigate an anecdotal link between damming the Mapé River in Cameroon and the emergence of BU in the Health Districts bordering Lake Bankim, the impoundment created by the Mapé dam. We used bacterial population genomics and molecular dating to find compelling support for a 2000 *M*. *ulcerans* introduction event that followed about 10 years after the filling of the newly created impoundment in 1988. We compared the genomic reconstructions with high-resolution satellite imagery to investigate what major environmental alterations might have driven the emergence of the new focus.

## Author summary

Buruli ulcer is a destructive skin and soft tissue infection caused by *Mycobacterium ulcerans*. Although the disease is not fatal, the infection can often leave patients with significant cosmetic and functional damage to limbs. Currently, one of the major hurdles facing Buruli ulcer control is our incomplete understanding of the factors leading to the emergence of disease in a particular region. In this report we investigate an anecdotal link between the damming of the Mapé River in Cameroon and the emergence of Buruli ulcer in the Health Districts bordering the impoundment created by the Mapé dam. We compare the genome sequences of *M*. *ulcerans* isolates recovered from regional Buruli ulcer patients that were identified in a previous molecular epidemiology study. Additionally, we investigate historic satellite imagery to quantify changes in land cover use that followed damming the river. The appearance of Buruli ulcer in the region was found to follow about ten years after the 1988 damming of the Mapé River, supporting the idea that alterations to landscape hydrology can increase BU incidence. While this temporal association does not infer causation, this research helps define the ecological risk factors linked to the spread of BU.

## Introduction

Buruli ulcer (BU) is a neglected tropical disease caused by infection of subcutaneous tissues with the pathogen *Mycobacterium ulcerans*. BU has a focal epidemiology and occurs mainly in certain areas of West and Central Africa, but has also been reported in the Americas, Oceania, and Asia [1]. Within endemic countries, disease foci are known to primarily occur around low-lying rural marshes, wetlands, and riverine areas [2]. As proximity to these slow flowing riparian and lentic habitats is a well-established risk factor for *M*. *ulcerans* infection [3], and as human-to-human transmission is very uncommon, it is generally believed that *M*. *ulcerans* is an environmental mycobacterium that can infect humans through introduction via a micro-trauma of the skin [4, 5]. However, the exact mode of BU transmission is unknown, and the definitive understanding of the factors leading to the focal emergence of BU disease in humans in a particular region is yet to be established. In various reports, changes to landscape hydrology have been linked to BU disease outbreaks in communities located in close proximity of such disturbances [6]. As such, increased BU incidence has been associated with natural and human-associated disturbances like exceptional flooding after unusually heavy rainfall, the implementation of irrigation systems, alluvial mining operations, and the creation of impoundments and wetlands [7]. Even though the anecdotal associations between disturbed water bodies and increased BU incidence is commonplace in the BU literature, evidence in support of a causal association has remained lacking.

**Fig 1:**
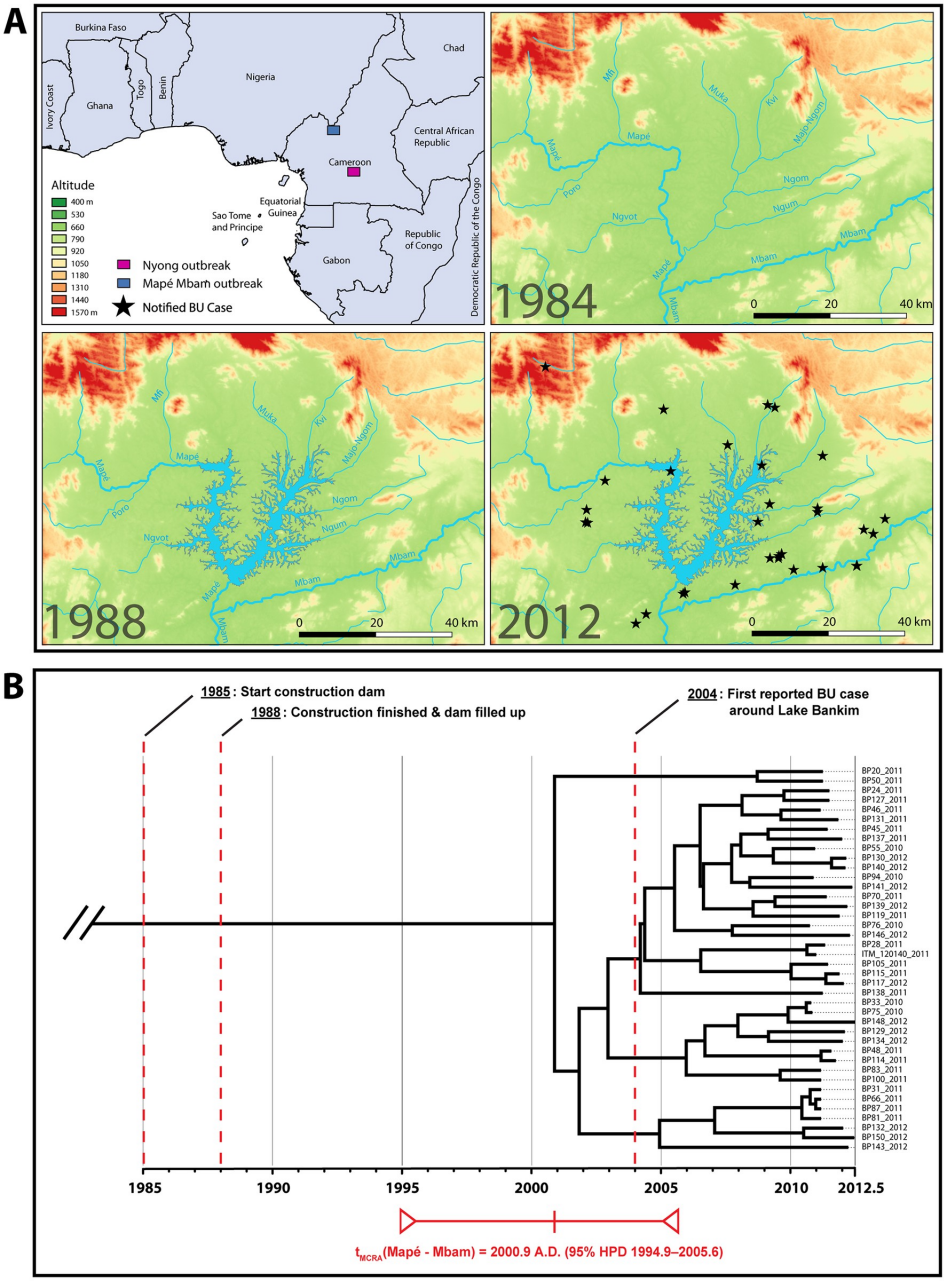
Panel A: Physical geography of the Mapé-Mbam outbreak area through time. The blue box in the first panel indicates the location of the study area. The GPS positions of the domiciles of the 40 laboratory confirmed BU cases included in the study are rendered as black stars. The river layer (Mapé River and its tributaries) was digitized from the declassified Soviet military topographic map b32-18 (scale 1:200k). The elevation data was obtained from the Shuttle Radar Topography Mission (SRTM). Vector map data of African administrative boundaries was obtained from Natural Earth. The panel was visualized using QGIS v.2.18.13. Panel B: Bayesian maximum clade credibility phylogeny for 40 *M*. *ulcerans* isolates from the Mapé-Mbam outbreak area. The tree was visualized in Figtree v1.4.3 [46]. Discussed key dates are annotated on the time-tree.

The first BU-endemic area of Cameroon was described in 1969 around the Nyong River, between the cities of Ayos and Akonolinga [8–10]. Thirty-five years later, in 2004, an additional BU endemic area was identified in the Bankim Health District (HD) of Cameroon (Fig 1A) [11, 12]. Whole genome analyses showed that two distinct *M*. *ulcerans* clades are found in these two endemic areas [13]. The Bankim HD is located more than 400km away from the Nyong River area. The environment around the Bankim BU focus is dominated by Lake Bankim, a 236 km^2^ impoundment created by the damming of the Mapé River close to its mouth in the Mbam River. This damming project was implemented in the 1980s to both meet Cameroon’s increasing electricity requirements and regulate the flow of the Sanaga River downstream of the Mapé and Mbam River during the dry season [14]. The infrastructure works effectively started in 1985 and by July 1988 the structures were completed and the dam filled (Fig 1A) [14]. Studies have hinted to the possible role of the Mapé storage dam construction in the appearance of BU in the Bankim HD [11]. The local population has also suspected that the damming of the Mapé river and the creation of an impoundment has led to an increase in BU incidence [15].

Molecular-dating methods can be applied to DNA sequence data to estimate the timing of evolutionary events of interest [16]. These methods measure genetic divergence between DNA sequences and then impute the time elapsed since the sequences diverged from a common ancestor. Molecular-dating methods have recently been successfully applied to *M*. *ulcerans* outbreaks in Africa [17, 18] and Oceania [19], revealing insights into reconstructed historical evolutionary trajectories. Here we used the same approaches by comparing the genome sequences of *M*. *ulcerans* isolates recovered from BU patients from the Mapé-Mbam basins, to test the hypothesis that the arrival of BU around Bankim followed the construction of the Mapé storage dam. To achieve this we re-analyze whole genome sequencing (WGS) data that originates from a 2010–2012 molecular epidemiology study which identified BU patients in the Bankim disease focus through an exhaustive cross-sectional house-by-house survey [13, 15]. After we identified support for an introduction event that followed the creation of the artificial lake, we used high-resolution satellite imagery to quantify the changes in land cover use that followed damming the river, and to better understand what major environmental alterations might have driven the emergence of the new disease focus.

## Methods

### Ethics statement

Ethical clearance for the collection and processing of the original samples was obtained from the Cameroon National Ethics Committee (N°041/CNE/DNM/09, N°006/CNE/SE/2010, and N°172/CNE/SE/2011), the Ethics Committee of the Heidelberg University Hospital, Germany (N°ISRCTN72102977) and the Ethics Committee of Basel (EKBB, reference n. 53/11). Participation was voluntary and all patients who participated in the study or their legal guardian provided written informed consent.

### Study area

The Mapé-Mbam BU endemic study area is located approximately 250 km north of the Cameroonian capital Yaoundé and covers primarily the Bankim HD but also parts of the neighboring HDs of Malantouen, Nwa, and Yoko (S1 Fig). Lake Bankim itself is encompassed by the HDs of Bankim and Malantouen (S1 Fig). The lake was created by damming the Mapé River, a sub-tributary of the Mbam river which is itself part of the Sanaga river basin and forms the border between the Bankim and the Yoko HDs (S1 Fig).

An extensive Bankim HD house-by-house survey (48,962 individuals from 9,344 households visited), followed by continued surveillance, identified a total of 88 laboratory confirmed BU cases between 2010 and 2012 [15]. The sequence data [13] we re-analyze here originates from the disease isolates collected from patients recruited in this survey. We therefore refer to Bratschi MW *et*. *al* 2013 for additional details on both the original survey and the epidemiology of the Mapé basin disease focus.

### Data acquisition

In total, 39 Illumina HiSeq 2000, 100 bp paired-end read sets were obtained from the NCBI Sequence Read Archive (SRA—BioProject accession PRJEB4025). We took care only to include a single isolate per patient in the panel. In addition, 157 other lineage Africa I (Mu_A1) genomes which represent the breadth of the West and Central African *M*. *ulcerans* diversity (described in [17]) were included to provide appropriate genetic context for interpreting the diversity of *M*. *ulcerans* from the Mapé river basin. One of the isolates of this panel, ITM_120140, originated from the Bankim BU disease focus, totaling the number of isolates from the focus up to 40. Detailed sequencing statistics for all isolates of the panel are provided in S1 Table.

### Read alignment and SNP detection

Read alignment and SNP detection were performed using the Snippy v4.2.1 pipeline [20]. The Burrows-Wheeler Aligner (BWA) v0.7.17 [21] was used to map filtered and trimmed read-pairs to the *M*. *ulcerans* Agy99 bacterial reference chromosome (Genbank: CP000325). After read mapping to *M*. *ulcerans* Agy99, average read depths were determined with SAMtools v1.9 [22] and are summarized for all isolates in S1 Table. SNPs were subsequently identified using the variant caller FreeBayes v1.2.0 [23], with the minimum number of reads covering a position to be considered as 10, the minimum mapping quality to accept in variant calling as 60, the minimum quality a nucleotide needs to be used in variant calling as 13, and the minimum proportion of reads which must differ from the reference at a position as 0.9. Snippy-core was used to pool all identified SNP positions called in at least one isolate and interrogate all isolates of the panel at that position. We used the snippy-core—mask auto option to exclude any SNPs in repetitive regions of the reference genome (655 kb/5.63 Mb, i.e. 11,64% of Agy99) which are largely made up of ISE elements (IS*2404* and IS*2606*). As previous recombination analysis [17] couldn’t detect any recombination events in African *M*. *ulcerans* we weren’t required to mask SNPs in recombination segments. As such a multiple sequence alignment of “core SNPs” was generated (S1 File).

### Bayesian phylogenetic analysis

We used BEAST2 v2.5.1 [24] to date evolutionary events and produce a time-tree of *M*. *ulcerans* from the Mapé river basin. The analyzed panel included both the 40 isolates from the studied basin and the set of 156 other *M*. *ulcerans* Mu_A1 isolates. BEAUti xml’s were manually modified to specify the number of invariant sites in the genome. An uncorrelated log-normal relaxed molecular clock [25] was used with a coalescent Extended Bayesian Skyline Plot (EBSP) tree prior [26] and bModelTest [27] to infer a genome scale Mapé river basin *M*. *ulcerans* time-tree with tip-dates defined as the year of isolation (S1 Table). The models we selected here are based on previous Bayesian phylogenetic studies into African *M*. *ulcerans* [17, 18] which employed model selection using path sampling [28] to compare the performance of various competing demographic and clock models. Analysis was performed in BEAST2 using a total of 5 independent chains of 800 million generations, with samples taken every 80,000 MCMC generations. Refer to S2 File for the xml file used in the BEAST2 analysis. Log files were inspected in Tracer v1.6 [29] for convergence, proper mixing, and to see whether the chain length produced an effective sample size (ESS) for all parameters larger than 400, indicating sufficient sampling. LogCombiner v2.5.1 [24] was then used to combine log and tree files of the independent BEAST2 runs, after having removed a 30% burn-in from each run. Thus, parameter medians and 95% highest posterior density (HPD) intervals were estimated from 35,000 sampled MCMC generations. To ensure prior parameters were not over-constraining the calculations, the entire analysis was also run while sampling only from the prior, and the resulting parameter distributions were compared in Tracer. TreeAnnotator v2.5.1 [24] was used to summarize the posterior sample of time-trees in order to as to produce a maximum clade credibility tree with the posterior estimates of node heights visualized on it.

We performed a date-randomization permutation test to assess the validity of the temporal signal in the sequence data [30]. Tip dates were randomly reshuffled to the sequences in 40 additional BEAST2 runs which otherwise used identical model settings as in the analysis of the real tip date data. This reshuffled “null set” of tip date and sequence correlations was then compared with the substitution rate estimate of the genuine tip date and sequence correlation. We furthermore tested the robustness of our findings using different coalescent demographic models (constant population size / Bayesian Skyline Plot (BSP)).

### Maximum likelihood phylogenetic analysis

We first performed jModelTest v2.1.10 [31] substitution model selection among the 11 basic substitution schemes, including models with unequal frequencies, gamma rate variation and a proportion of invariable sites. The GTR model with no rate heterogeneity was selected as one of the best-fitting models using both the AICc and BIC criteria. Maximum likelihood (ML) phylogenies were then estimated 100 times from the core SNP alignment (S1 File) using RAxML v 8.2.12 [32] under the GTR model (no rate heterogeneity) with likelihood calculation correction for ascertainment bias using the Lewis method (-m ASC_GTRCAT -V). The tree with the highest likelihood across the 100 runs was selected. We performed 10,000 rapid bootstrap analyses to assess support for the inferred ML phylogeny.

### Remote sensing

QGIS v.2.18.13 [33] was used to generate the figures of the geographical distribution of BU cases and land cover uses around Lake Bankim. High resolution satellite imagery from the study area was acquired from Landsat earth-observing satellites by courtesy of the U.S. Geological Survey (USGS). This imagery was used to extract land cover types and detect land cover changes between the year 1984 (Landsat 5: acquisition date 29/11/1984) and 2015 (Landsat 8: acquisition date 21/12/2015) using the Semi-Automatic Classification Plugin v5.3.6.1 for QGIS. Additionally, all available imagery from the study region acquired between 1984 and 2015 by Landsat 5, 6, 7 and 8 was used to determine Bankim Lake’s hydrodynamics over time [34].

## Results

### Comparative genomics and phylogenetic analysis

Illumina sequence reads of the Mapé-Mbam *M*. *ulcerans* isolate panel were aligned to the *M*. *ulcerans* Agy99 bacterial reference chromosome and, after excluding repetitive elements and small insertion-deletions (indels), we detected a total of 95 SNPs with approximately 1 SNP per 52,380 bp (0.002% nucleotide divergence). The Mapé-Mbam isolates had an average pairwise SNPΔ of 8 (SD = 3). A total of 35 clones (unique genomes) could be discerned among the 40 isolates in the panel. All 40 Mapé-Mbam isolates corresponded to lineage Africa I (Mu_A1), and the uncommon lineage Africa II (Mu_A2) was not identified in the study region [17]. This might be a result of our limited panel size though, as Mu_A2 was found to be relatively rare in better sampled BU hotspots like the southern Beninese disease focus (2.0%, 14 Mu_A2 / 684 total Beninese strains) and the Congo river basin focus (0.6%, 1/179) [18].

A Bayesian maximum clade credibility time-tree was estimated from the alignment of the extended African isolate panel (S2 Fig). Additionally, from the same alignment, a ML phylogeny was inferred which closely matched the topology and relative branch lengths of the time-tree (S4 Fig). Within both phylogenies, all Mapé-Mbam isolates formed a strongly supported (posterior probability of 1) monophyletic group (S2 Fig). Additionally, this Mapé-Mbam group was more related to a clade of 135 *M*. *ulcerans* isolates sampled from all over the African continent than to the 17 isolates from the Nyong River basin that formed a separate monophyletic group (S2 Fig). As a result, our analysis reconfirmed the previously described [13] distinct spatial clustering of *M*. *ulcerans* from the different Cameroonian BU foci, indicating strong geographical restrictions on *M*. *ulcerans* dispersal.

Across all 196 African *M*. *ulcerans* isolates as a whole, the inferred mean substitution rate was 1.07E-7 substitutions per site, per year (95% HPD interval [5.56E-8-2.01E-7]). The Bayesian analysis indicated *M*. *ulcerans* isolates from the Mapé-Mbam outbreak shared a common ancestor (t_MCRA_(Mapé-Mbam)) in 2000.9 CE (95% HPD 1994.9–2005.6) (Fig 1B). A similar time-scale was also observed under the alternative coalescent models, constant population size [1999.4 CE (95% HPD 1994.1–2003.6)] and BSP 2002.9 CE (95% HPD 1996.8–2007.5), indicating that the age estimate is robust to the selected tree prior. We also assessed the reliability of our estimated timescales by conducting a date-randomization permutation test. Bayesian 95% HPD intervals using the real sampling times did not overlap with those from any of the randomizations (S3 Fig).

To investigate the possible origin of the Mapé-Mbam outbreak we compared the genomes from the Mapé-Mbam basin to those from across other West and Central African BU endemic countries (S2 Fig). The closest match (40 SNP difference) obtained was to isolates ITM_071924 and ITM_071925, sampled 1160 km away from a single patient originating from the Republic of Congo (Kouilou Department). The Bankim outbreak isolates shared a common ancestor with these Congolese isolates in 1971.7 CE (95% HPD 1949.6–1991.8). Interestingly, the second and third best matches represented isolates from two different states in bordering Nigeria. Isolate ITM_121474 (Cross river state—Ogoja LGA—280 km) had a 73 SNP difference with the Mapé-Mbam clade and shared a common ancestor with it in 1943.8 CE (95% HPD 1909.1–1975.0). Isolate ITM_070386 (Anambra State—Ayamelum LGA– 480 km) was slightly more diverged (157 SNPs) from the Mapé-Mbam clade and shared an ancestor with it in 1860.2 CE (95% HPD 1793.5–1921.9).

### Remote sensing

**Fig 2:**
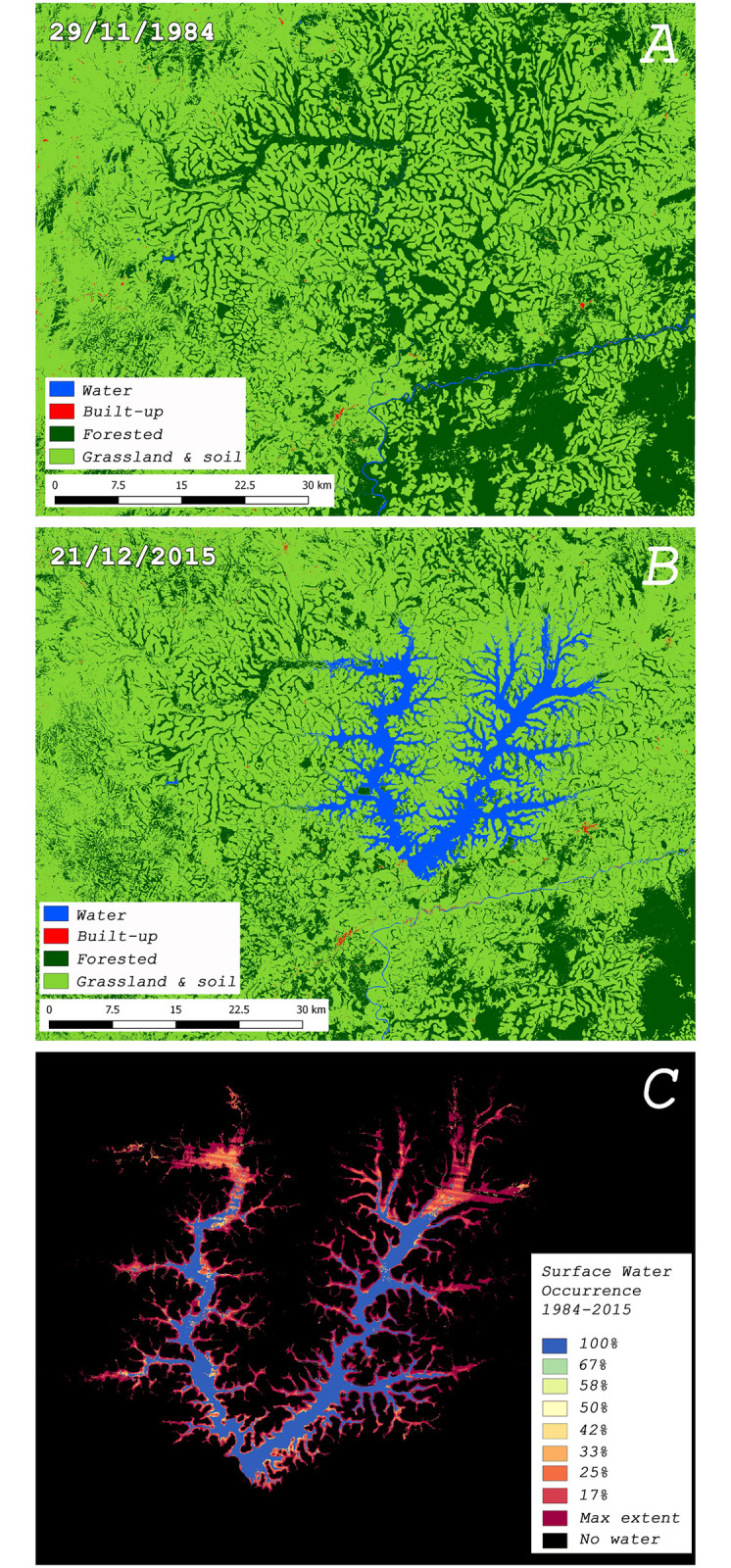
Panels A & B: Land cover use around the Mapé-Mbam outbreak area during 1984 and 2015. Land cover classification was performed using a supervised approach. Panel C: surface water occurrence on all obtainable Landsat imagery between 1984 and 2015; expressed as a percentage of the available water observations over time. This overview captures both the intra and inter-annual variability and changes in surface water occurrence in order to provide a consistent characterization of the “water dynamic” over time. High resolution satellite imagery from the study area was acquired from Landsat earth-observing satellites by courtesy of the U.S. Geological Survey (USGS). This imagery was used to extract land cover types and detect land cover changes between the year 1984 (Landsat 5: acquisition date 29/11/1984) and 2015 (Landsat 8: acquisition date 21/12/2015) using the Semi-Automatic Classification Plugin v5.3.6.1. The figure was visualized using QGIS v.2.18.13.

We used high-resolution satellite imagery of the 4974 km^2^ study area to measure changes in land cover use before and after damming the Mapé River. The main environmental effect of the Mapé dam infrastructure project was the flooding of 158 km^2^ of forest and 78 km^2^ of grassland & open soil. Additionally, filling the impoundment created swamps and meadows along the estimated 1074 km long lake shoreline. Finally, a total of 333 km^2^ of forest was cleared and 2 km^2^ of additional built-up area was created in the environment around Lake Bankim (Fig 2A and 2B). We also observed considerable intra- and inter-annual variability in the total surface area of the lake over the 31 years of Landsat observations (1984–2015). The lake can flood up to 154 km^2^ of additional land when it is filled from its average lake surface area (82km^2^; 50–100% water occurrence) to the maximum observed water extent (max extent-236km^2^-100% water occurrence) (Fig 2C).

## Discussion

Whether BU is a recently recognized, preexisting disease or rather newly emerging in the Mapé-Mbam basin is hard to establish, since no records of skin diseases in the region exist that are older than the surveys performed in 2004 and 2010 [12, 15]. Oral reports on the occurrence of BU cases in the area prior to 2004 cannot be verified, since no clinical records of such cases exist, and laboratory reconfirmation of clinical diagnosis has not been sought. This is why we investigated the timing of the *M*. *ulcerans* introduction event in the Mapé-Mbam outbreak area using a Bayesian coalescent approach that co-estimated both evolutionary rates and dates of divergence. The deepest node on our timed phylogeny (Fig 1B), corresponding to the time of origin of *M*. *ulcerans* strains sampled in the Mapé-Mbam basin, had a median age of 11.6 years (95% HPD 17.6–6.8) relative to the most recent sampling time (2012.5). Therefore, the analysis indicated that *M*. *ulcerans* had likely been introduced in the Mapé-Mbam basin in 2000.9 CE (95% HPD 1994.9–2005.7). The year 2000.9 is recent compared to the reported introduction times of *M*. *ulcerans* in a number of other well-sampled African BU areas associated with the Nyong (Cameroon), Oueme (Benin), and Congo (Democratic Republic of Congo—Republic of Congo) River basins, all of which temporally aligned with the 19th and early 20th centuries [17]. Furthermore, the Bayesian HPD credible interval associated with the Bankim BU introduction event only encompasses a 11-year period which is relatively narrow compared to the estimates of the other aforementioned African introductions all of which spanned close to a hundred years. As a result, the temporal analysis provides compelling support for the hypothesis that an *M*. *ulcerans* introduction event followed about 10 years after damming of the Mapé River and the subsequent filling of the newly created impoundment in 1988 (Fig 1B). These observations are supported by a 2008 study in the Ivory coast that suggested an association between BU incidence and large storage dams [35].

The timing of the 2000.9 introduction event also allowed us to infer that the Mapé-Mbam basin had been endemic for an estimated four years before the first BU patients were reported in 2004. This estimate is similar to those in a recent report from Australia that also compared the temporal emergence of *M*. *ulcerans* in three specific outbreak regions with the timing of the first reported BU cases in these regions [19]. Authors of this report hypothesized that *M*. *ulcerans* might require an estimated seven to nine-year period of environmental and/or zoonotic expansion before being able to spill over and cause disease in humans [19].

To investigate the possible origin of the Mapé-Mbam BU outbreak we compared the Mapé-Mbam *M*. *ulcerans* genomes to a panel of genomes that represents the breadth of the West and Central African *M*. *ulcerans* genetic diversity [17]. Even though we found phylogenetic evidence for a possible link with isolates from the Republic of Congo and western Nigeria, it is not possible to specify these as the origin of the Mapé-Mbam basin BU focus given the limited isolates sampled from both countries. As a consequence, the route of introduction and immediate origin of *M*. *ulcerans* introduced to the Mapé-Mbam basin remains to be established.

BU endemic areas on the African continent are generally found where the wet equatorial tropical rainforest predominates; this includes the longer identified BU endemic focus around the Nyong River. The environment of the Mapé-Mbam basin differs substantially from the Nyong River area, as it is dominated by tropical deciduous forest-savanna. We tried to use multi-temporal remote-sensing imagery to investigate how the emergence of BU might be a consequence of marked changes in the environment caused by filling of Lake Bankim. Before the start of the infrastructure works for the Bankim dam, the area largely consisted of “gallery” forests that formed as corridors along rivers and projected into the surrounding open grasslands and shrublands that were otherwise sparsely treed (Fig 2A). The main environmental effects of the construction project were the flooding of a vast areas of grass- and forestland, and the creation of swamps and meadows close to the margins of the reservoir. Studying the temporal water dynamics of the lake also revealed substantial seasonal and inter-annual fluctuations in its total surface area. This means that the lake frequently submerges considerable areas surrounding the lake’s average shoreline. Additionally, along the immediate margins of the lake, we observed substantial amounts of deforestation brought about mostly by slash-and-burn agricultural activities. Interpretation of these observations should be made with caution and in the context of all biological information about the disease, as we cannot be certain these changes in land use facilitated the transmission of *M*. *ulcerans*. A good example of this is the creation of the Lake Volta in Ghana, one of the largest man-made lakes in the world; even though BU is prevalent in various neighboring river basins [17] the disease is hardly ever found in communities situated along the borders of lake Volta [36].

The damming project had a considerable socio-economic impact: 345 people from 32 villages were displaced and resettled, three new villages developed spontaneously, the region was electrified, a network of 138 km of farm roads was constructed, and the increased irrigation potential greatly enhanced the production of agricultural cash crops such as corn, banana, beans, rice, cassava and groundnuts [14, 37]. The creation of an impoundment also formed an emerging economic area around fishing and the processing and selling of fish products [14].

The consequences of damming the Mapé River included direct impacts to the chemical and physical properties of the river and its environment which resulted in the creation of novel ecological niches characterized by water stagnation, increased light levels, higher water temperatures, and lower oxygen levels. Some authors have hypothesized that these major changes in physio-chemical abiotic factors can create environmental conditions that allow for the persistence and/or growth of *M*. *ulcerans* [7]. However, analyzing the geographic distribution of laboratory confirmed BU cases in the Bankim HD revealed that these cases were more associated with agricultural activities at the Mbam river rather than with the artificial Mapé reservoir itself [15]. As a result, only a few BU patients were living in the immediate proximity of the reservoir which challenges the importance of direct contact with the lake in establishing new infections. This observation does however not exclude the possibility of an indirect effect of the damming of the Mapé river on the spread of BU in the area as large dams influence BU incidence not only at the reservoir site, but also downstream. In French Guiana, the construction of the Petit-Saut Dam on the Sinnamary River in 1994 was followed by a significant decrease in the amount of observed BU cases in the inhabited zone 40 km downstream from the impoundment [38]. Authors hypothesized that damming the river substantially modified the ecological functioning of the floodplain, which led to a reduced abundance of *M*. *ulcerans* or its reservoir in or along the river. Bratschi *et al*. [39] detected *M*. *ulcerans* DNA at a few village water sites at some distance from Lake Bankim. While the inferred concentration of bacteria in all positive samples was very low, at a particular location in a shallow water hole, detritus remained positive over a period of more than two years. The frequency of positive sample occurrence was low, and definite evidence for the presence of viable *M*. *ulcerans* in the samples was lacking owing to the challenge of culturing the slow growing mycobacterium from non-clinical, environmental sources [40]. As a result, this comprehensive environmental sampling survey, similar to other studies [41, 42], indicated that *M*. *ulcerans* DNA can be present in the environment, although the significance of this phenomenon remains unclear with respect to the epidemiology of BU.

Damming the Mapé River undoubtedly also had impacts on the biological properties of the river. Dams are known to potentially cause an expansion in the number and range of habitats of vectors of waterborne infectious diseases such as schistosomiasis (freshwater snail vector) [43]; malaria and bancroftosis (mosquito vector) [44]; and onchocerciasis (fly vector) [45]. Currently though, there is no compelling experimental evidence for biological transmission of *M*. *ulcerans* via a particular vector in Africa, even though various authors have brought forward potential vector species.

Hydropower is the most mature, non-polluting, renewable energy technology, with a low cost of operation. As Cameroon has the second highest hydropower potential in Central Africa after the DRC, it is the main source of energy in Cameroon. Although hydrological dams are important for water and energy supply, flood management, and irrigation, it is also established they can influence increases in communicable and vector-borne diseases [45]. Here we used a molecular-dating method for the first time to link a particular BU-disease outbreak with a major anthropogenic hydromorphological alteration to an aquatic landscape. Our analysis provides compelling support for an *M*. *ulcerans* introduction event that closely followed damming the Mapé River. It is tempting to posit damming the Mapé River might have at least indirectly created the slow flowing lentic habitats that are a well-established risk factor for *M*. *ulcerans* infection. In conclusion, we have discovered a strong temporal association between construction of a dam that created a large inundation and the emergence of BU. While association does not infer causation, this research helps define the ecological risk factors linked to the spread of BU.

## Supporting information

S1 Fig Regional HDs of the Mapé-Mbam outbreak area. The administrative borders of HDs were obtained from the Health Information System (SNIS) of the Ministry of Public health of Cameroon. The GPS positions of the domiciles of the 40 laboratory confirmed BU cases included in the study are rendered as black stars. The elevation data was obtained from the Shuttle Radar Topography Mission (SRTM). The figure was visualized using QGIS v.2.18.13.
(TIF)

S2 Fig Bayesian time-tree for 40 *M*. *ulcerans* isolates from the Mapé-Mbam outbreak area and 156 other Mu_A1 African genomes. Branches are color coded according to their branch specific substitution rate (legend at top). Geographically localized clonal expansions associated with two particular hydrological basins (Mapé-Mbam, and Nyong) are highlighted with boxes. The tree was visualized in Figtree v1.4.3 [46].
(TIF)

S3 Fig Date-randomization test. A comparison of Bayesian estimates of substitution rates for real and randomized tip dates. Squares and the circle represent median estimates, while bars indicate values of the 95% HPD interval. The estimate obtained using the real tip date associations (circle) is shown on the far right, while estimates from random associations (squares) are shown on the left. Sequence data is considered to have strong temporal structure when the substitution rate estimate obtained using the real tip-dates is not contained within the HPD intervals of rate estimates of the randomizations.
(TIF)

S4 Fig Maximum likelihood tree for 40 *M*. *ulcera*ns isolates from the Mapé-Mbam outbreak area and 156 other Mu_A1 African genomes. The tree was visualized in Figtree v1.4.3 [46].
(TIF)

S1 Table Isolate panel with sequencing statistics. (XLSX)

S1 File Multiple sequence alignment of core SNPs used in both the Bayesian and ML phylogenetic analysis. (FASTA)

S2 File BEAST2 xml file used in the Bayesian phylogenetic analysis. (XML)
