# Supplementary figures and images for: Introduction of Mycobacterium ulcerans disease in the Bankim Health District of Cameroon follows damming of the Mapé River
Source: PLoS Negl Trop Dis. 2020 Sep 4;14(9):e0008501. doi: 10.1371/journal.pntd.0008501 (PMC7473558; doi:10.1371/journal.pntd.0008501)

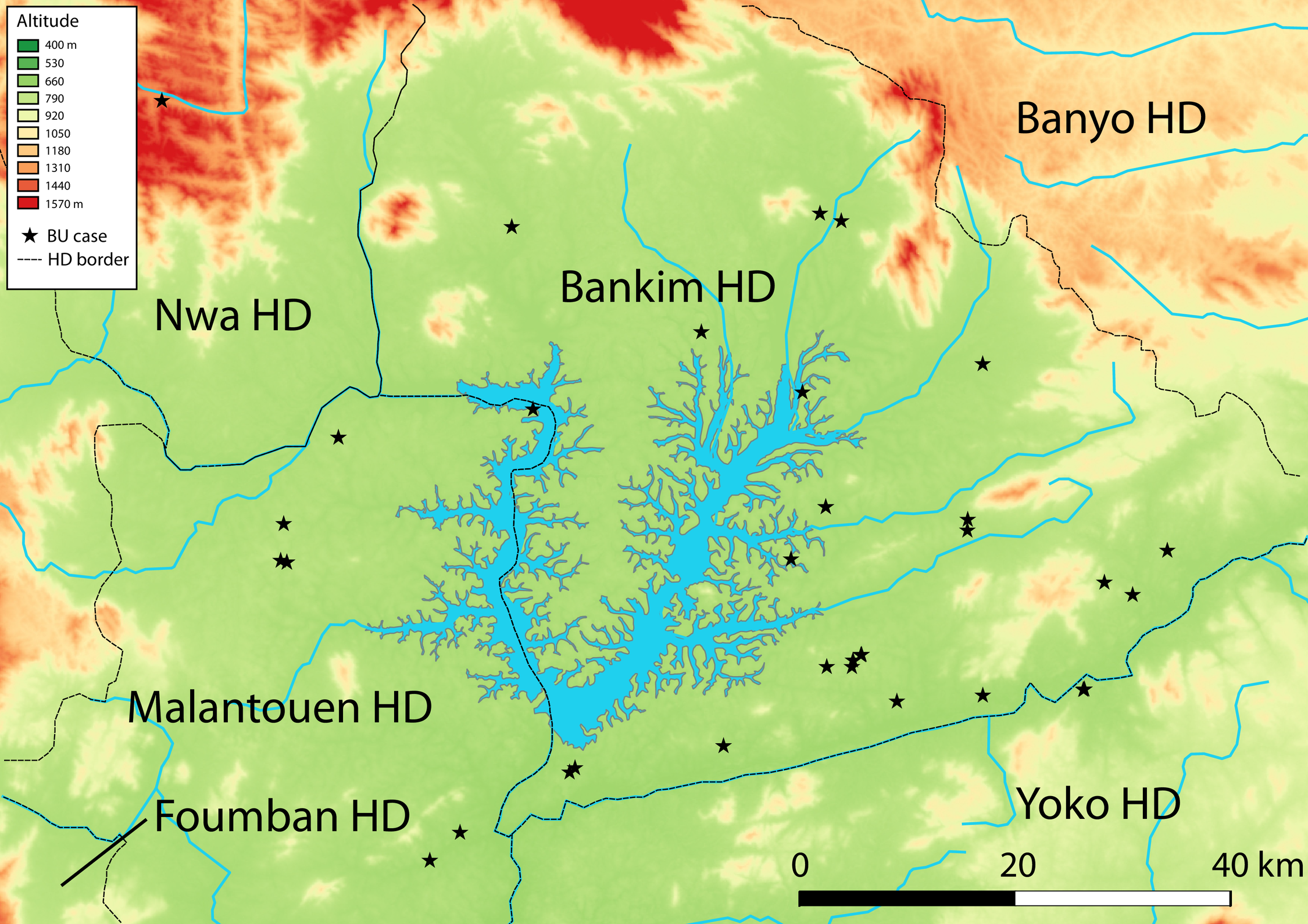

Supplement: S1 Fig — The administrative borders of HDs were obtained from the Health Information System (SNIS) of the Ministry of Public health of Cameroon. The GPS positions of the domiciles of the 40 laboratory confirmed BU cases included in the study are rendered as black stars. The elevation data was obtained from the Shuttle Radar Topography Mission (SRTM). The figure was visualized using QGIS v.2.18.13. (TIF) [file pntd.0008501.s001.tif]

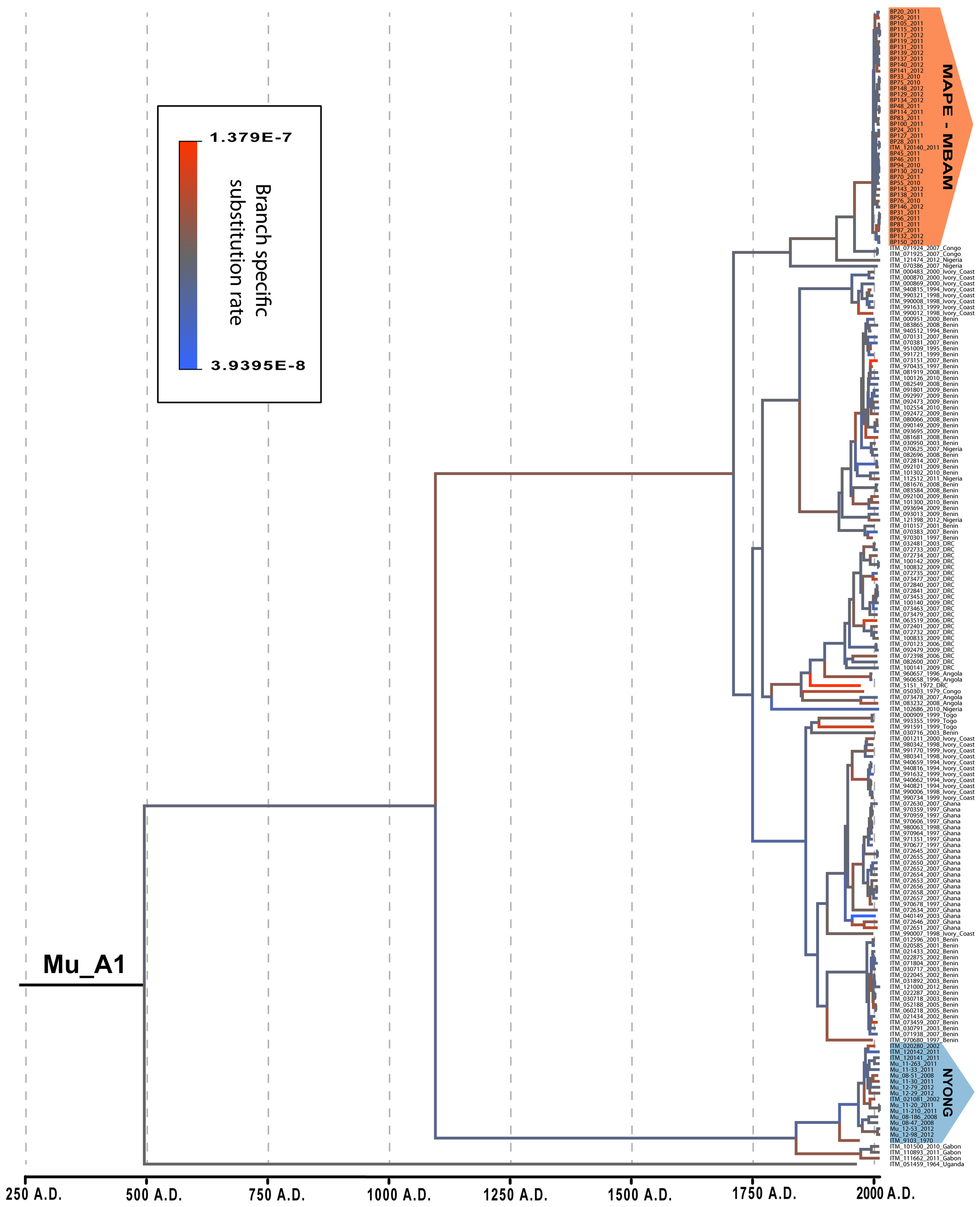

Supplement: S2 Fig — Branches are color coded according to their branch specific substitution rate (legend at top). Geographically localized clonal expansions associated with two particular hydrological basins (Mapé-Mbam, and Nyong) are highlighted with boxes. The tree was visualized in Figtree v1.4.3 [46]. (TIF) [file pntd.0008501.s002.tif]

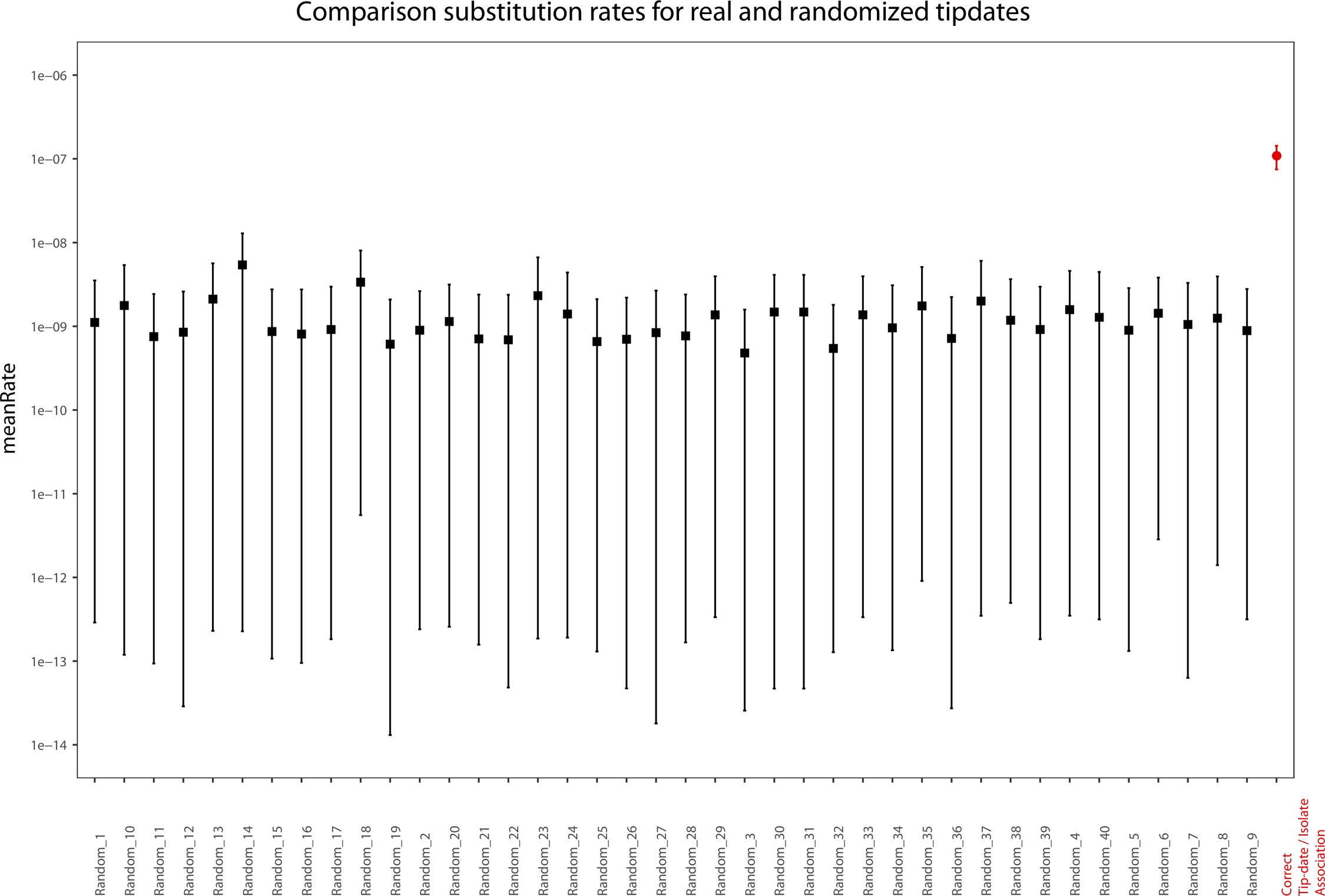

Supplement: S3 Fig — A comparison of Bayesian estimates of substitution rates for real and randomized tip dates. Squares and the circle represent median estimates, while bars indicate values of the 95% HPD interval. The estimate obtained using the real tip date associations (circle) is shown on the far right, while estimates from random associations (squares) are shown on the left. Sequence data is considered to have strong temporal structure when the substitution rate estimate obtained using the real tip-dates is not contained within the HPD intervals of rate estimates of the randomizations. (TIF) [file pntd.0008501.s003.tif]

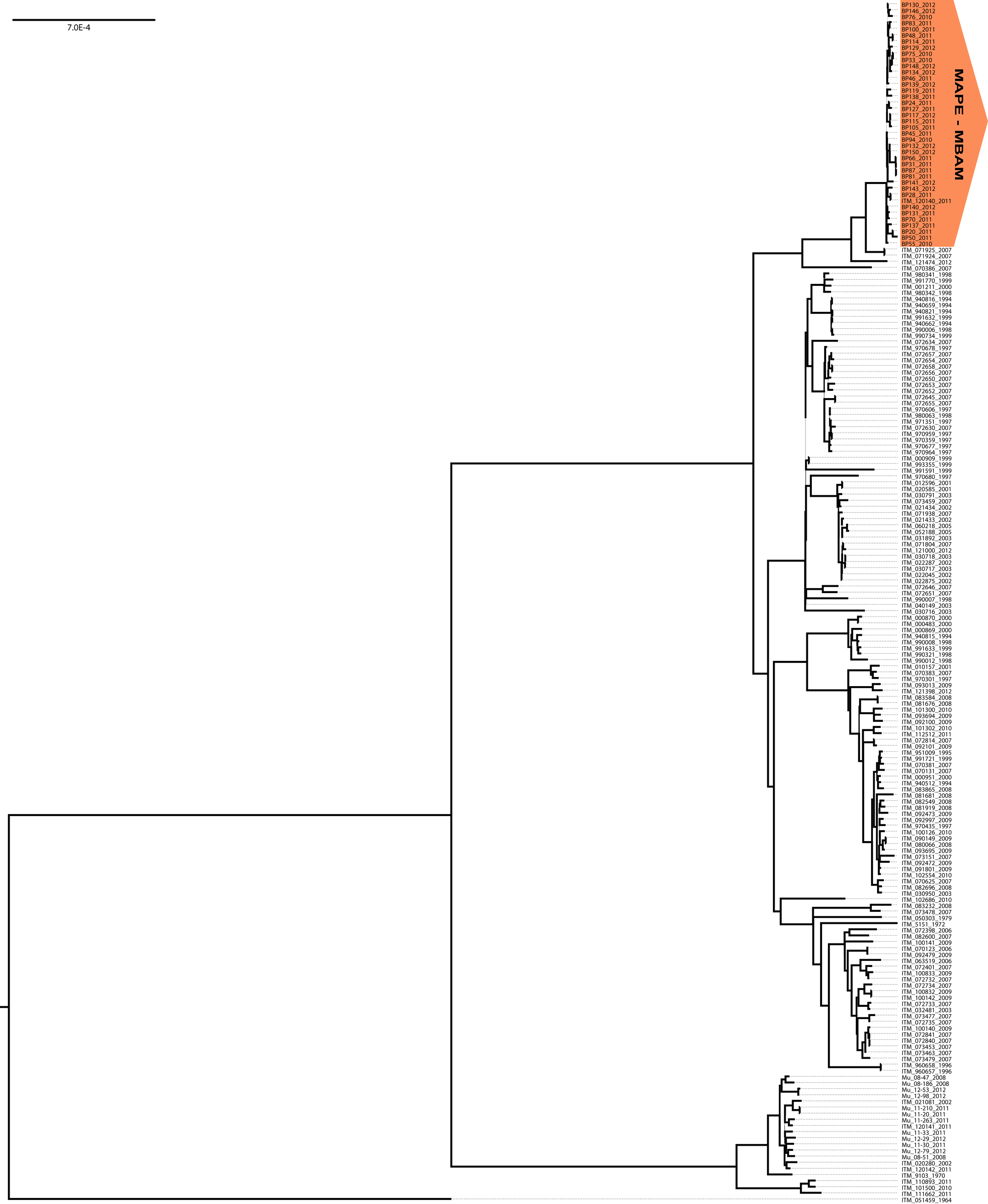

Supplement: S4 Fig — The tree was visualized in Figtree v1.4.3 [46]. (TIF) [file pntd.0008501.s004.tif]
